# A Distributed Data-Gathering Protocol Using AUV in Underwater Sensor Networks

**DOI:** 10.3390/s150819331

**Published:** 2015-08-06

**Authors:** Jawaad Ullah Khan, Ho-Shin Cho

**Affiliations:** Department of Electronic Engineering, Kyungpook National University, Daegu 702-701, Korea; E-Mail: jawaad_khan@ee.knu.ac.kr

**Keywords:** underwater sensor network, autonomous underwater vehicle, TDMA, clustering, Voronoi region

## Abstract

In this paper, we propose a distributed data-gathering scheme using an autonomous underwater vehicle (AUV) working as a mobile sink to gather data from a randomly distributed underwater sensor network where sensor nodes are clustered around several cluster headers. Unlike conventional data-gathering schemes where the AUV visits either every node or every cluster header, the proposed scheme allows the AUV to visit some selected nodes named path-nodes in a way that reduces the overall transmission power of the sensor nodes. Monte Carlo simulations are performed to investigate the performance of the proposed scheme compared with several preexisting techniques employing the AUV in terms of total amount of energy consumption, standard deviation of each node’s energy consumption, latency to gather data at a sink, and controlling overhead. Simulation results show that the proposed scheme not only reduces the total energy consumption but also distributes the energy consumption more uniformly over the network, thereby increasing the lifetime of the network.

## 1. Introduction and Motivation

In the last few decades, researchers have shown tremendous interest in the deployment of sensor fields called underwater sensor networks (UWSNs) for various applications related to environmental monitoring, tactical surveillance, and reconnaissance data acquisition in oceanic fields. Most of the sensor nodes are interconnected through wireless links in such networks. These wireless links use acoustic signals because the propagation of radio or optical signals is severely affected by a large amount of absorption and scattering loss. However, acoustic signaling imposes many design challenges on communication protocol owing to high bit error rate, limited bandwidth, and long propagation delays. Under such poor channel conditions, high transmission power is necessarily used, and accordingly undesirable interference may occur widely over the network. This introduces a large amount of overhead in the form of retransmissions. Therefore, sensor nodes are forced to communicate with each other over a short distance as a possible way to combat the highly erroneous channels and limit the interference. Accordingly, multihop transmission techniques have been preferred as a data-gathering scheme to collect data distributed over an area into a pre-determined location known as a sink [1].

In a multihop transmission scenario, some sensor nodes act as a relay for other nodes located at a distance from the sink. It is observed that relay nodes consume most of their energy in relaying, and consequently exhaust their energy resources much faster than the other nodes. These relay nodes become ineffective over a period of time owing to rapid energy depletion which eventually results in disconnections in a large network. This phenomenon is known as the energy-hole problem in a sensor network. Therefore, many data-relaying schemes have been proposed for the multihop UWSN in order to reduce such uneven energy consumption in addition to improving other network performance parameters [1,2,3]. However, application of these protocols requires excessive bandwidth and energy resources in case of a large-scaled network.

Domingo and Prior [4,5] proposed a clustering approach, where the sensor nodes forward data to a node called a cluster head (CH) in a single hop manner. Then, cluster heads employ multihop transmission technique to forward the data to the final destination, a sink. Although this data-relaying technique is an effective way to reduce the energy consumption in a large set of nodes, the issue of uniform energy consumption still remains unsolved for cluster heads. Thus, the need for using a mobile sink arises. The mobile sink can travel to neighborhoods to collect data so that sensor nodes may conserve energy by avoiding multihop and long-distance transmissions. The deep sea networking scenarios described in NorthEast Pacific Time-Series Undersea-Networked experiments (NEPTUNE) [6], Seatooth [7] and Subsea monitoring [8] are such examples, where an AUV visits several data gathering neighborhoods for data collections. In such cases, since the sensor nodes are not easily accessible for maintenance as compared to AUV and other surface elements like buoy, it is imperative to design an energy efficient data-gathering technique to prolong the lifetime of the sensor nodes.

In [9], it is shown that an autonomous underwater vehicle (AUV) working as a mobile sink can effectively reduce the transmission range of sensors, which leads to saving energy for sensor nodes during transmission. An AUV travels a specified path and stops at number of locations, called tour points to gather data. The neighborhood of a tour point is highly random in nature because of constantly changing environmental conditions, and therefore is called a probabilistic neighborhood. The AUV probes the probabilistic neighborhood to discover nodes during a specific time interval called a probe interval. 

After a probe interval, the AUV creates a communication schedule only for identified sensor nodes. The neighboring sensor nodes use a random access technique to reply to the AUV-transmitted probe signal during the probe interval. It is highly probable that some reply packets transmitted by sensor nodes may be lost owing to collisions or an adverse channel. This could lead to the failure of a network to detect crucial events that may be catastrophic in certain applications. In addition, the AUV has to wait for all information to be retrieved from the neighborhood before it moves to the next neighborhood. Thus, this approach increases the AUV’s touring time which has an adverse effect on AUV operational costs. Moreover, owing to the probabilistic nature of the neighborhood, it is very difficult to achieve uniform energy consumption over the entire neighborhood.

Similarly, in [10] the authors considered a polling scheme for an AUV to communicate neighboring sensor nodes at a tour point. In such a data-gathering approach, the probabilistic neighborhood, where the probability of successful communication is low, may result in many retransmissions. These retransmission overheads cause additional costs in term of excessive resource consumption in addition to AUV operational costs. 

In [11], the authors analyzed a heterogeneous underwater network scenario, where sensor nodes are categorized based on their functionalities. In such an approach, a special fixed node called a head node takes the responsibility of gathering data from a neighborhood. In this scheme, head nodes, which are distributed over the network, collect data from respective neighborhoods and forward the data to an AUV that is taking a data-gathering tour. However, the authors have not discussed how these head nodes may be placed or selected in a network to form a number of data gathering neighborhoods that deal with issues such as non-uniform energy consumption at ordinary nodes during transmission to head nodes or the rapid depletion of energy at head nodes during an AUV data-gathering tour. 

As a result, the approach of using fixed relaying nodes may be highly prone to failure owing to uneven depletion of the nodes’ energy in certain coverage areas, which may directly affect overall network performance. It is evident that the prior information on the probabilistic neighborhood results in deterministic neighborhood, which may increase the efficiency of the AUV probing interval. Therefore, a framework is to be developed that may result in deterministic neighborhoods for the AUV at the tour path, which is still an open issue that needs to be addressed. In addition, the framework should also be able to handle the selection of each relay-node for a neighborhood in such a way that uniform energy consumption may take place over the network.

The designing of such a framework that achieves a deterministic neighborhood for AUV data gathering and which may also meet the requirement of uniform energy consumption is a challenging task owing to adverse channel conditions and absence of global information on energy consumption. In the proposed scheme, a node clustering technique is employed, which has been known [4,5] as a way to design a more deterministic neighborhood for AUV data collection on a tour point. The proposed scheme uses the hybrid multiple access protocol of time division multiple access (TDMA) and code division multiple access (CDMA).

In the proposed scheme, an AUV visits some identified locations that act as a temporary sink. To do this, the AUV travels the network deployment region in a predefined lawn-mower pattern to deliver network-partitioning information to sensor nodes. Based on the partitioning information, the entire network is organized into a number of clusters so that each contains a cluster head (CH). Then, the CH further divides the cluster into several subclusters and nominates a sensor node called a path-node (PN) for each subcluster to collect local data from the member-nodes (MN) and reduce the impact of unequal inter-nodal transmission distances. The CH disseminates the information on the list of PNs and requests the PNs to collect data from their respective subclusters. After partitioning the network, the AUV initiates a data-gathering tour with the probe interval in a predetermined neighborhood and communicates with the CH to acquire the list of PNs. Then, the AUV visits each PN to collect available data.

The proposed data-gathering scheme, which is named AUV-visits-PN (AUV-PN), is evaluated and compared in terms of total amount of energy consumption, the standard deviation of each node’s energy consumption, latency to gather the data at a sink, and controlling overhead with the following possible alternatives for data gathering:

1. AUV-visits-CH (AUV-CH): In this scheme, the CH collects data from MNs and acts as a data relaying node for the cluster. During a data-gathering tour, the AUV only visits the CH to collect the data.
2. Domingo routing protocol [4]: This is a data-gathering approach where no mobile sink is employed. A network is partitioned into a number of clusters, each of which contains a CH that collects the data from respective MNs and forwards it to the sink in a single or multihop manner.

The main contributions of this paper can be summarized as follows:

1. An approach for designing the deterministic AUV data-gathering neighborhoods is presented, keeping in mind the absence of global information.
2. The proposed scheme selects relay nodes from the set of already deployed sensor nodes, thus relieving the scheme from special installation requirements [11].
3. The performance of the proposed scheme is compared with the existing schemes in terms of standard deviation of energy consumption, latency, and overhead.

The preliminary version of this work was presented in a prior conference paper [12]. The present paper extends the conference version with additional results related to energy consumption during the network partitioning phase, protocol overhead, and distribution of energy consumption in the network. The rest of the paper is organized as follows. In Section 2, an acoustic link budget is presented. In Section 3, system description is given. Then, Section 4 presents the operation of the proposed AUV-based data gathering protocol. After that, protocol performance evaluation is presented in Section 5. Finally, Section 6 concludes this paper.

## 2. Acoustic Link Budget

The passive sonar equation [13] is used to analyze the energy consumption over an acoustic link. If a sensor node transmits an acoustic signal with power level *SL* (in dB re µPa), then the received signal to noise ratio (*SNR*) per bit at a receiving node can be expressed as

(1) SNR=SL−TL−NL−DI     (dB)

where *TL*, *NL*, and *DI* are the transmission loss over the acoustic link, the noise power level at a receiving node and the directivity index of transmitting antenna, respectively. It is assumed that sensor nodes are equipped with omni-directional antennas, and therefore *DI* is considered to be zero. Similarly, the transmission loss with unit normalizing constant *A*_0_, which includes fixed losses over distance *d* (meter), is taken as [14]

(2) $$TL=A_{0}+10\times k\times \log_{\;10}(d)+\alpha (f)\times d\times 10^{-3}$$

where the spreading factor *k*, which defines the geometry of propagation, is equal to 1.5 for practical scenario and α(*f*) is the absorption coefficient in dB/km. For frequency *f* (kHz), the absorption coefficient α(*f*) is expressed empirically using the Thorpe formula as [15],

(3) $$\alpha (f)=\frac{0.11f^{2}}{1+f^{2}}+\frac{44f^{2}}{4100+f^{2}}+2.75\times 10^{-4}\times f^{2}+0.003$$

Underwater turbulence, shipping activity, wave, and thermal noise are the main sources of noise in an underwater channel. However, for practical applications, the noise power level *NL*, in dB re µPa per Hz, can be approximated as [15]

(4) NL=50−18×log(f)

Similarly, a node requires power *P* (watt) to transmit an acoustic signal with intensity *I* at a distance of 1 m in the direction of the receiver, which can be expressed as [16]

(5) $$P=2\pi \times H\times I=2\pi \times H\times 10^{SL/10}\times 0.67\times 10^{-18}$$

where *H* is the depth in meters. By solving Equations (1) and (5), the expression for the received *SNR* can be written as

(6) $$SNR=10\times \log(P)-10\times \log(2\pi \times H\times 0.67\times 10^{-18})-TL-NL$$

It is observed that acoustic channel is prone to the multipath effect. There are two well-known causes of multipath formation in an acoustic channel [17]:

1. Sound reflection at any objects or bottom and surface of ocean
2. Sound refraction in water owing to spatial variability of sound speed

Therefore, a transmitted acoustic signal is found to be severely faded by multipath in an underwater environment. This fading of acoustic signal is a random process that can be modeled as a Rayleigh fading. If a binary-phase-shift-keying (BPSK) modulated acoustic signal is transmitted, then the average bit error probability *P_b_* is expressed as [18]

(7) $$P_{b}=\frac{1}{2}-\frac{1}{2}\sqrt{\frac{10^{SNR/10}}{1+10^{SNR/10}}}$$

Accordingly, packet error rate (PER) for packet length *L*(bits) can be computed as

(8) $$PER=1-{\left(1-P_{b}\right)}^{L}$$

## 3. System Description

### 3.1. Basic Assumptions

It is assumed that *N* numbers of identical underwater acoustic sensor nodes are submerged in a given oceanic region to monitor environmental events and record meaningful ecological changes for applications described in [6,7,8]. These sensor nodes are scarce in resources such as power, data storage, data processing and sensing range capabilities. These sensor nodes are also equipped with communication modules that have limited range. Sensor nodes can adjust their transmission power to maintain one hop communication link. Each sensor node is aware of its geographical location and its unique identification number which is called an address. 

It is also considered that an AUV is available for exploring the designated area. The AUV uses onboard guidance and navigational tools to traverse a specific path called a tour path. It is assumed that the AUV achieves error free localization using techniques based on inertial navigation system (INS) and acoustic navigation as described in [19,20]. The deployment information of the sensor nodes is known to the AUV. Therefore, the AUV can navigate using this information to stop near a sensor node for data gathering. It is also assumed that the AUV has mechanism to adjust its speed and achieve a certain depth in a designated area. The AUV is also capable of establishing a communication link with a range much longer than normal nodes. Similarly, the AUV can also process and store a large amount of data for later usage.

### 3.2. Network Architecture

**Figure 1:**
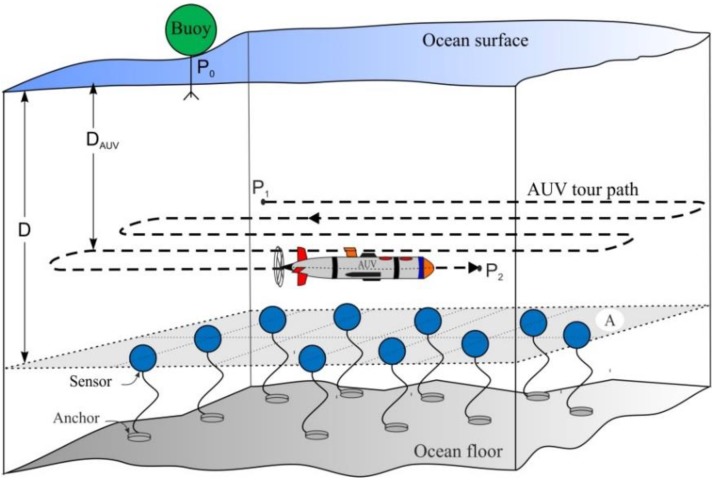
Network architecture.

A UWSN described in [4,21] is considered, where sensor nodes are uniformly distributed in two-dimensional plane *A* denoted in Figure 1 at a depth *D* of a given three-dimensional region *R*^3^. These sensor nodes are considered static as they are anchored to the ocean floor and there is a surface buoy, which acts as a sink located at position **P_0_**. The AUV is operating at a constant depth *D_AUV_ ˂ D* with an average velocity *v*.

The plane *A* is partitioned into several regions named clusters in order to gather data in a more efficient way. For this purpose, the AUV first computes the Voronoi generator point [22] cluster, and then executes a network partitioning tour (NPT) to broadcast the Voronoi generator points which are used by sensor nodes to identify the cluster to which they belong.

After clustering, member nodes (MNs) of each cluster select a delegate node called a cluster head (CH) that is responsible for the following tasks:

1. To further divide a cluster into several subclusters.
2. To select a primary data-gathering node named a path-node (PN) in every subcluster, which relays the gathered data to AUV.
3. To disseminate the list of PNs to share throughout the cluster.

After subclustering, the AUV executes another tour, called a data-gathering tour (DGT), to visit every PN of which addresses are informed by the CH. The detail of this procedure is given in Section 4.

### 3.3. Data Gathering Procedure

The data is gathered in three steps: first, from MN to PN (MN→PN); next, from PN to AUV (PN→AUV); and finally, from AUV to sink (AUV→sink). The MN→PN is carried out constantly except during PN→AUV. During DGT, the AUV travels to each cluster and obtains the list of PNs from the CH. Then, the AUV visits every PN in the order of the list, to take the data-gathering step PN→AUV. After visiting the last cluster, the AUV returns to the original tour-starting point **P_1_**, and then ends the DGT with the step AUV→sink.

### 3.4. Multiple Access Scheme

**Figure 2:**
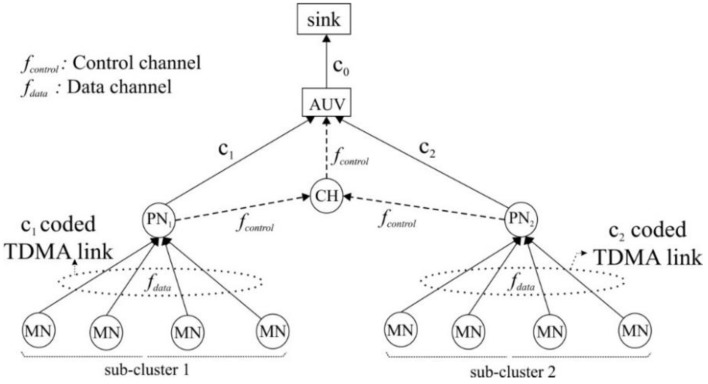
Medium-access scheme.

The frequency band is divided into two parts: one is for data (*f_data_*) and the other is for control (*f_control_*). The control channel is shared by all network nodes by means of a contention-based protocol such as MACA-U, MACA-UPT, or ROPA [23,24]. By contrast, the data channel is exclusively allocated to nodes by means of contention-free protocol such as TDMA and CDMA. In much of the recent research, TDMA is found to be an effective way to meet intra-cluster communication requirements [5,25]. Thus, we adopt TDMA for multiplexing the link MN→PN, where PN is in-charge of time-slot allocation, as shown in Figure 2.

CDMA is employed to escape the interference between subclusters. A different orthogonal code is assigned to each subcluster by the CH [26,27]. That is, the TDMA multiplexed data from MNs are additionally coded by the assigned orthogonal code. The links of CH→AUV and PN→CH are used only to exchange of the control information; therefore, they share the contention-based control channel of *f_control_*. The link AUV→sink also uses CDMA with a dedicated orthogonal code. 

## 4. Operation of Proposed Scheme

**Figure 3:**
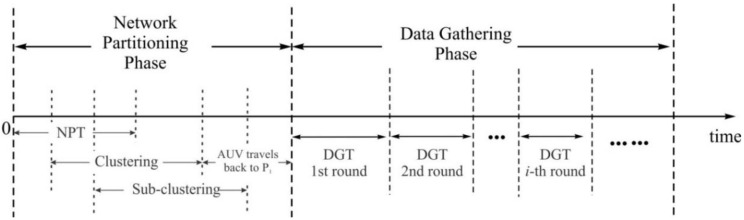
Timeline of the proposed scheme.

The proposed scheme runs through two phases, as shown in Figure 3. The scheme begins with a network-partitioning phase (NP_Phase), where an AUV computes network-partitioning information such as Voronoi generator points and CDMA codes, and then executes an NPT to broadcast the information. The NPT starts from a point **P_1_** at which a communication link to the sink is available.

During the NPT, sensor nodes identify the cluster to which they belong, based on the network- partitioning information and accordingly elect a CH. Then, the CH further partitions the cluster into several subclusters and locates a PN in every subcluster based on the geographical distribution of traffic requirements by the MNs in a way that the overall energy consumption for data-gathering in MN→PN is minimized. After the NPT, the proposed scheme enters a data-gathering phase (DG_Phase), where the DGT repeats rounds until a new network partitioning is required. During every round, data is gathered from all distributed MNs to one sink through three steps: MN→PN, PN→AUV and AUV→sink. During DG_phase, the PN keeps gathering data from MNs except when the PN is working for PN→AUV.

### 4.1. Network Partitioning Phase

**Figure 4:**
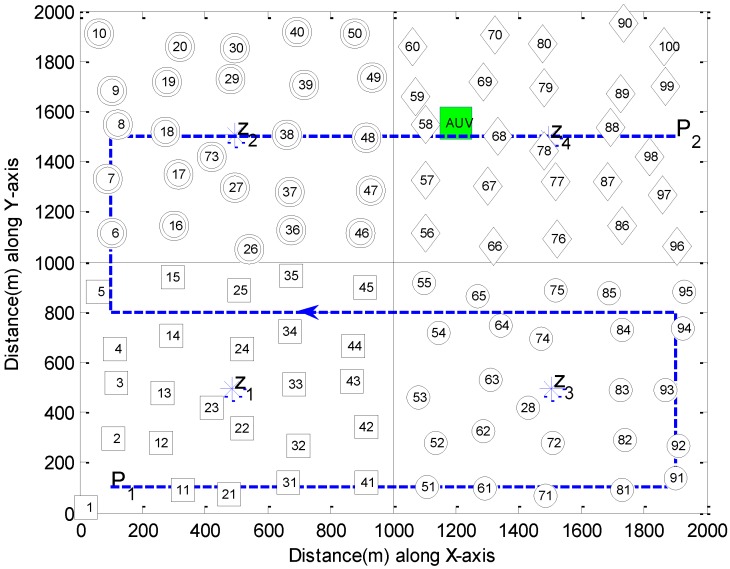
UWSN with four clusters after NPT.

First, the AUV employs the concept of a Voronoi region [28] to compute generator points which are used by sensor nodes to identify the cluster to which they belong. Suppose that region *i* of plane *A* has *N_i_* nodes of which locations are denoted by the two-dimensional vector *x_k_* ,*k* = 1, 2, 3,...,*N_i_*. Then, generator point *z_i_* for the region is defined by [22,29]:

(9) $$z_{i}=\frac{1}{N_{i}}\sum_{k=1}^{N_{i`}}x_{k}\text{ where }i=1,2,3...,K$$

which is subject to the operation of vector summation and scalar multiplication to the vector. Similarly, Voronoi region *V_i_* associated with generator point *z_i_*, can be defined as

(10) $$V_{i}=\left\{x_{k}\in A\;|\left\|x_{k}-z_{i}\right\|<\left\|x_{k}-z_{q}\right\|\right\}$$

where ||.|| represents the Euclidean distance. Here, the Voronoi region corresponds to one cluster. Figure 4 shows an example of an NPT along the lawnmower pattern to partition an area of 4 km^2^ into four clusters separated by a dash-dot line. The sensor nodes with unique addresses are shown with distinct markers in each cluster. Similarly, generator points are depicted by asterisk (*) symbols (labeled as z*_i_*), and the AUV tour path during the NPT between points **P_1_** and **P_2_** is represented as a dashed line with the arrowhead pointing in the direction of motion of the AUV. Identifying the cluster, sensor nodes elect a CH by using advertisement and the cluster set-up phase of the LEACH protocol [28,30]. During this process, the CH obtains the information of MNs, including their addresses and locations. 

As a next step, CH further partitions the cluster into several subclusters using the Voronoi criteria of Equation (10) based on the location information of MNs, in the same way as clustering. Then, the CH selects a temporary PN for every subcluster and provides the information about associated MNs such as address and location. The CH also assigns an orthogonal code for CDMA between subclusters. After that, the temporary PN allocates TDMA time-slots to MNs, collects the information on the amount of traffic generated from each node, and then reports the information to the CH. Collecting the traffic information from all temporary PNs, the CH estimates the energy consumption associated with a traffic delivery from MNs to AUV in case a specific node *i* is selected as a PN, such as

(11) $$E_{MNs\to PN\to AUV}^{Delivery}(i)=\sum_{h=1,h\neq i}^{M}E_{h\to i}+E_{i\to AUV}$$

where *E_i→j_* is the amount of energy consumption for data delivery incurred between node *i* and node *j*, and *M* is the number of MNs within a subcluster. Comparing the energy of Equation (11) for all cases of *i* = 1,2,...*M*, CH selects the node *m* that will cost the minimum energy as

(12) $$m=\arg\;\underset{i}{\min}{\left(E_{MNs\to PN\to AUV}^{Delivery}(i)\right)}_{\;i=1}^{\;M}$$

Then, the CH announces the final PN selections using the information of associated MNs. The PN starts the data-gathering step MN→PN. 

**Figure 5:**
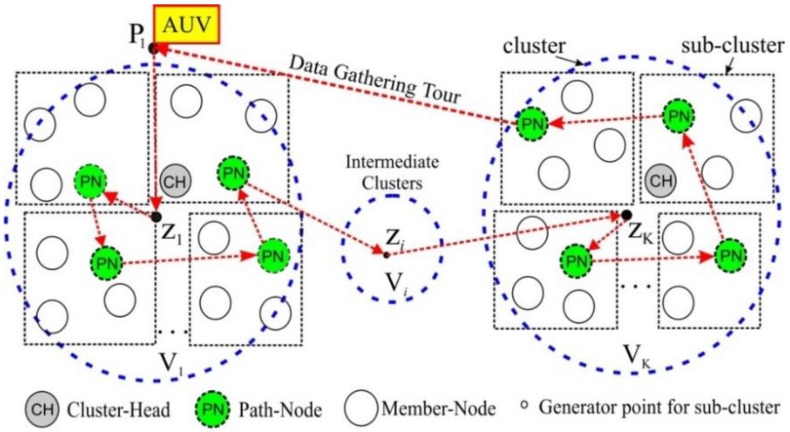
AUV data-gathering tour in UWSN.

### 4.2. Data Gathering Phase

After completing the NPT, the AUV stays at the ending point **P_2_** in Figure 4, until the last CH notifies the end of clustering. Then, the AUV returns to point **P_1_** to start a DGT. The AUV selects the nearest cluster as a first visit based on the information on generator points. Then, the AUV finds the associated CH in order to obtain the information required for data gathering, such as the list of PNs to visit and CDMA code being used on the MN→PN links. In most cases, the CH is located near the generator point. Since the AUV has knowledge of the generator points, it approaches each generator point as shown in Figure 5 and takes the well-known neighbor discovery procedure as proposed in [31] to search for the CH. Then, communicating with the CH through a control channel, the AUV acquires the aforementioned information.

**Figure 6:**
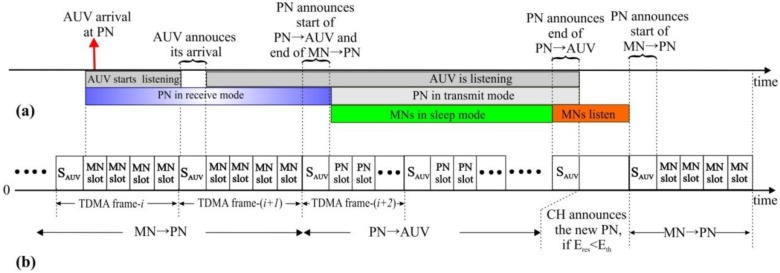
MN→PN and PN →AUV (**a**) communication related event (**b**) TDMA frame structure.

Figure 6 shows the frame structure of the MN→PN TDMA channel and the relevant events on the time axis. The TDMA channel is switched from MN→PN mode (PN in reception) to PN→AUV mode (PN in transmission) when the AUV arrives. The first time slot, named *S_AUV_* in every TDMA frame, is reserved for broadcasting of AUV-related signaling. Overhearing the TDMA channel to find out the *S_AUV_* slot, the AUV announces its arrival via *S_AUV_*.

Then, on the next *S_AUV_* slot, the PN also announces a mode change of the TDMA channel from MN→PN to PN→AUV and starts transmitting the data it has held for the AUV. The PN→AUV mode continues until the PN sends out all data. After that, the PN announces a mode change back from PN→AUV to MN→PN and also the residual energy via the *S_AUV_* slot. For the rest of the frame, a new PN could be selected by the CH if the residual energy of the current PN *E_res_* is less than a certain threshold level *E_th_*. During PN→AUV, MNs may go into sleep mode to save energy.

After completing the entire PN→AUV gathering for a given cluster, the AUV moves to the next cluster and follows the same procedure as described above until it reaches the last cluster. Then, the AUV returns back to location **P_1_** to execute the final step, AUV→sink, where a dedicated CDMA code is used to avoid interference to ongoing MN→PN communication in those surroundings. Completing AUV→sink, the AUV starts the next round of data gathering for the DG_Phase.

## 5. Performance Evaluation

In order to evaluate the performance of the proposed scheme, an event based simulation model for an UWSN with 100 sensor nodes uniformly distributed at a depth of 300 m, is developed in MATLAB. The acoustic channel attenuation is modeled with fixed channel losses *A_0_* of 30 dB [14], and channel noise is implemented as described in Section 2. In the simulation model, a target SNR of 20 dB is considered to achieve a packet error rate of 10^−3^ for binary phase shift keying (BPSK) modulation. In addition, the nominal speed of sound is assumed to be 1500 m/s. The transmission parameters of a WHOI Micro-Modem [14,32] are used for the acoustic channel model. Some of these parameters are listed in Table 1. We assume that the receiver is equipped with decision feedback equalizer and error-correction software that consumes additional energy of 500 mW. Furthermore, we also assume that a 128 MB high density RAM and a 32 GB microSD card are installed on every node to meet the temporary data storage requirements.

It is considered that an AUV moves with constant speed of 2 m/s at a depth of 250 m. For the network of 100 sensor nodes, 100 iterations of Lloyd’s algorithm [29] are run to compute uniformly distributed generator points. It is an iterative algorithm that calculates generator points using the knowledge of sensor nodes’ location. It starts with generation of randomly distributed generator points in a plane. In each iteration, a new set of generator points are calculated using Equation (9), after calculating Voronoi regions using Equation (10). The variance of nodes’ distances from the corresponding generator points and the number of iterations in the algorithm are used as the stopping criteria for running the algorithm. Regarding traffic generation at each node, the Poisson arrival process is employed.

**Table 1:** Simulation parameters.

Parameter	Value
Data Rate	2500 bps
DATA packet length *L_data_*	1024 bits
Control packet length *L_cont_*	64 bits
Initial energy *E_i_*	1000 J
Transmitter efficiency	0.25
Channel bandwidth	4 kHz
Center frequencies *f_control_*, and *f_data_*	25, 20 kHz
Max. transmit power	50 W
Receive and idle-listening power	300 mW (Micro modem) + 500 mW (Processing power)
Number of clusters	4
Energy threshold *E_th_* *=* 0.70 × *E_i_*	700 J
Vertical link (Relay node→AUV) distance	50 m

**Figure 7:**
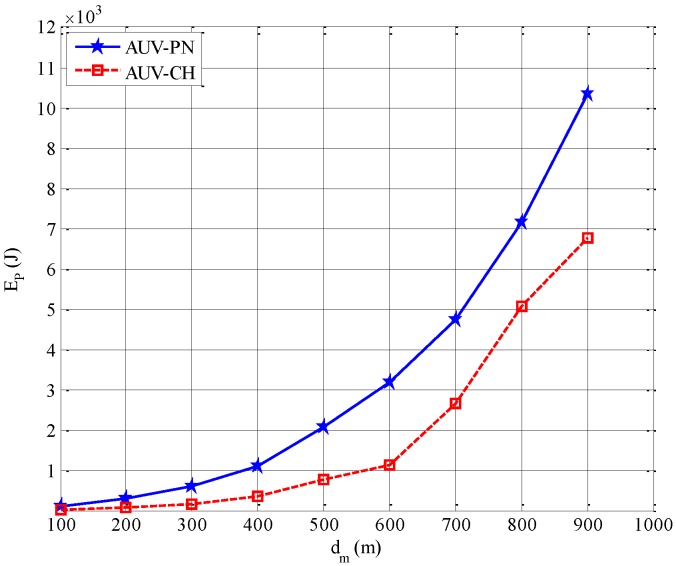
Energy consumption during NP_Phase, *E_P_*

Figure 7 shows, the energy consumed by all sensor nodes during NP_Phase with respect to mean inter-node distance *d_m_*. In AUV-CH, based on the assumption that clustering takes place in a similar way as described for AUV-PN, the energy consumption for network partitioning is calculated as

(13) $$E_{P}=E^{Clustering}+E^{AUV-NPT}$$

Where *E^Clustering^* and *E^AUV−NPT^* are the energy consumption during the cluster-head setup and the reception of partitioning information from the AUV, respectively. In AUV-PN, the energy consumption for network partitioning is calculated as

(14) $$E_{P}=E^{Clustering}+E^{AUV-NPT}+E^{sub-clustering}$$

where *E*^sub-clustering^ is the energy consumption associated with the additional process of subclustering and PNs selection after cluster-head setup. The effect of additional traffic generated in AUV-PN becomes more prominent for higher values of *d_m_*. Even though the proposed scheme has an additional component of energy consumption *E^sub-clustering^*, it will be shown that proposed scheme outperforms other candidates in terms of total energy consumption.

To compare the proposed scheme with conventional data gathering protocols, the Domingo routing protocol [4,5], which is a way of organizing the network into clusters and delivering the data by multihop transmission to reduce long-range transmission, is simulated along with the other AUV-employed schemes such as AUV-PN and AUV-CH. For the sake of fair comparative analysis for all protocols, the same network topology with an equal number of cluster has been kept, where *N = 100* nodes are uniformly distributed over an area of 1000 × 1000 m^2^.

Using Equations (13) and (14), the total energy consumption is obtained by

(15) $$E_{total}=E_{P}+E_{G}$$

where *E_G_* is the energy consumed by all sensor nodes during DG_Phase and is obtained by

(16) $$E_{G}=\sum_{i=1}^{N}E_{G-i}$$

where E_G-*i*_ is the energy consumption related data gathering of node *i* and is obtained differently according to whether the node is an MN or a relay node. If node *i* is an MN,

(17) $$E_{G-i}=E^{tx}+E^{slp}+E^{lstn}$$

where *E^tx^*, *E^slp^* and *E^lstn^* are energy consumptions for transmitting data to relay node, sleeping while PN works for PN→AUV, and idle-listening after the PN→AUV to hear, if any, announcement of new PN from CH, respectively. Here, we ignore the sleeping and idle-listening energies owing to very low power requirements during the sleeping process [10,17], and the relatively small idle-listening duration as compared with the transmission time. If node *i* is a relay node that would be the CH in AUV-CH or a PN in AUV-PN,

(18) $$E_{\text{G}-i}=E^{rx}+E^{fwd}$$

where *E^rx^* and *E^fwd^* are the energy consumptions for receiving data from the respective MNs and forwarding the data to AUV, respectively.

**Figure 8:**
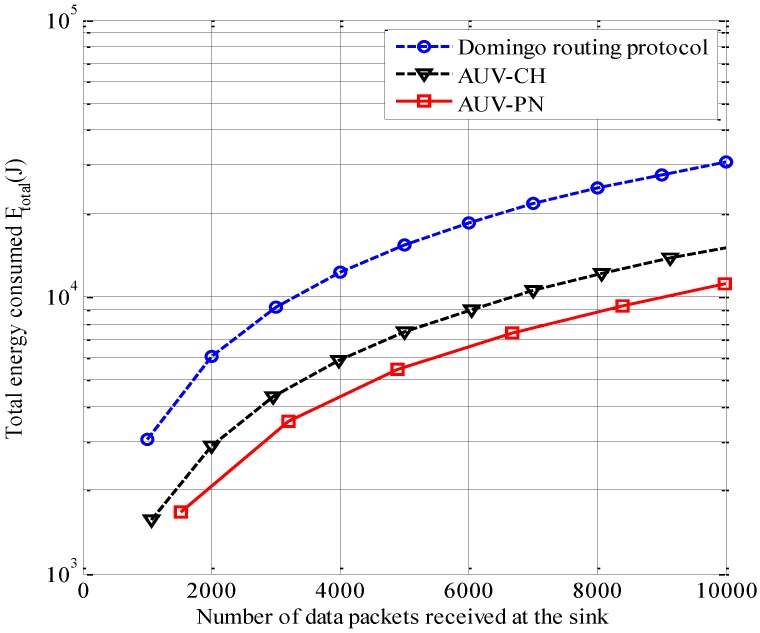
Total energy consumption *versus* the number of data packets gathered at the sink.

Figure 8 shows the total energy consumption *E_total_* for various numbers of data packets gathered at the sink. It is observed that the total energy consumption of AUV-PN is considerably less than those of AUV-CH and the Domingo routing protocol. This is primarily because AUV-PN has a larger number of relaying nodes than AUV-CH and uses single-hop transmission with a short distance, unlike the Domingo routing protocol, which uses multihop transmission with a longer distance. The larger number of relay nodes results in a shorter distance between MNs and the relay nodes, and therefore saves more energy.

**Figure 9:**
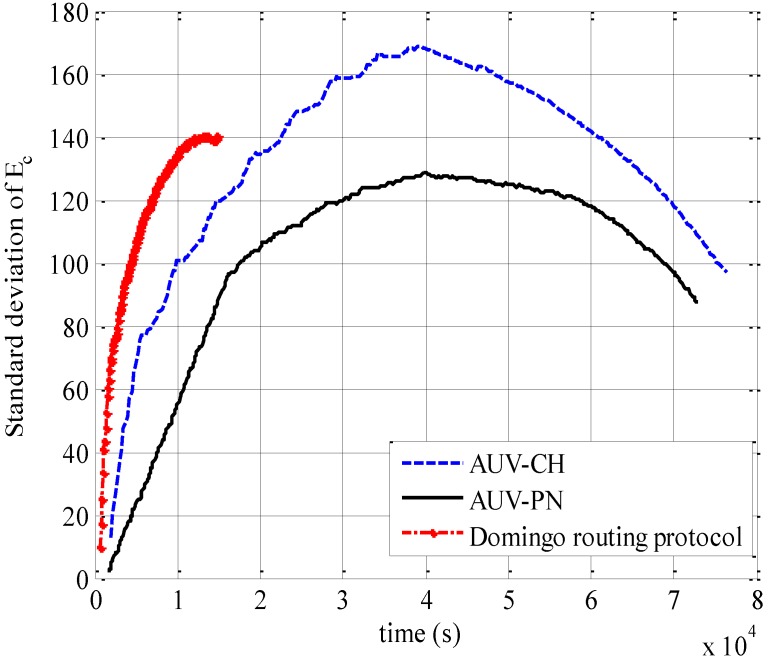
Standard deviation of energy consumption.

In addition to the amount of energy consumption, how evenly each node spends energy is necessarily considered, especially in order to augment the lifetime of the network. Thus, we examine system performance in terms of the standard deviation of energy consumption of each node, regardless of MNs and relay nodes in Figure 9. At the beginning, the standard deviation increases because the energy consumption is concentrated to several relay nodes such as PN and CH. However, as the relay nodes are replaced with new ones, the increase in standard deviation slows down and reaches a certain peak point where approximately half of the nodes have served as relay nodes. Then, the standard deviation starts to decrease as further changes in relay nodes take place. 

It is also observed that AUV-PN has the smallest standard deviation compared with AUV-CH and the Domingo routing protocol. This is because AUV-PN has a large number of relay nodes, each associated with a smaller data-gathering neighborhood as compared with AUV-CH, which results in a low number of data packets being received at the relay nodes and also being subsequently transmitted to the AUV. While in comparison with the Domingo routing protocol, AUV-PN does not require multihop transmission and thus avoids the transmission of unequal number of data packets between the relay nodes. On the other hand, the simulation for the Domingo routing protocol stops earlier than others because all MNs in the clusters near to the sink exhaust their energy beyond a threshold that we set up as a simulation ending point, much earlier. 

**Figure 10:**
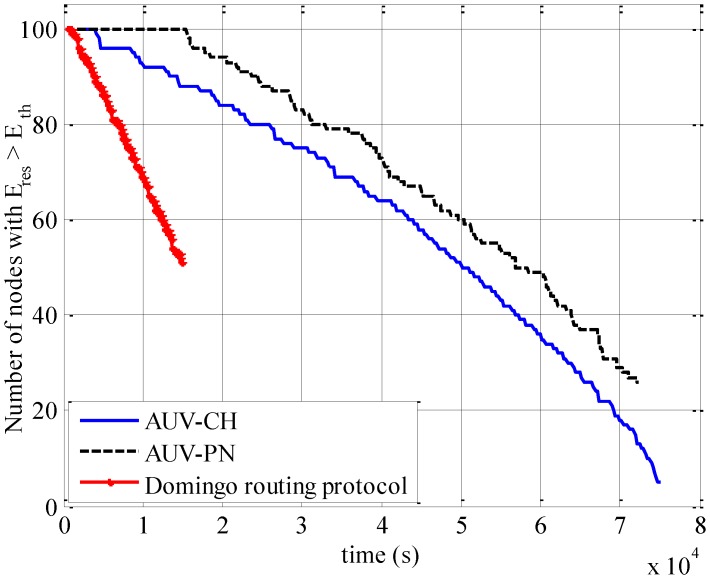
Number of nodes with residual energy E_res_ > E_th_.

To further elaborate on the impact of unequal energy consumption, we show the number of nodes whose residual energy *E_re_*_s_ is greater than threshold *E_th_* as function of time in Figure 10. It can be observed that the depletion of energy resources takes place at much lower rate in AUV-PN than for AUV-CH. This occurs because of the increased number of data-gathering neighborhoods in AUV-PN as compared with AUV-CH. As an example, it can be seen that 40 nodes deplete their energy resources beyond the threshold limit after the elapse of 4.4 × 10^4^ s for AUV-CH, while the same number of nodes exhaust their energy resources after the elapse of 5 × 10^4^ s for AUV-PN.

The improved residual energy performance can also be attributed to the number of transmissions from the relay nodes. For AUV-PN, the number of transmissions from the relay-nodes is much less than for those for the AUV-CH. This results in much lower energy consumption at the relay node. Therefore, the change in relay node occurs at a much slower rate than in the AUV-CH case. The Domingo routing protocol has a larger depletion rate of residual energy as compared with the AUV-based approaches; this is a result of the increase in the rate of energy consumption for nodes near the sink. Thus, it can be concluded that the AUV-PN approach conserves energy resources more efficiently than the AUV-CH approach and the Domingo routing protocol.

**Figure 11:**
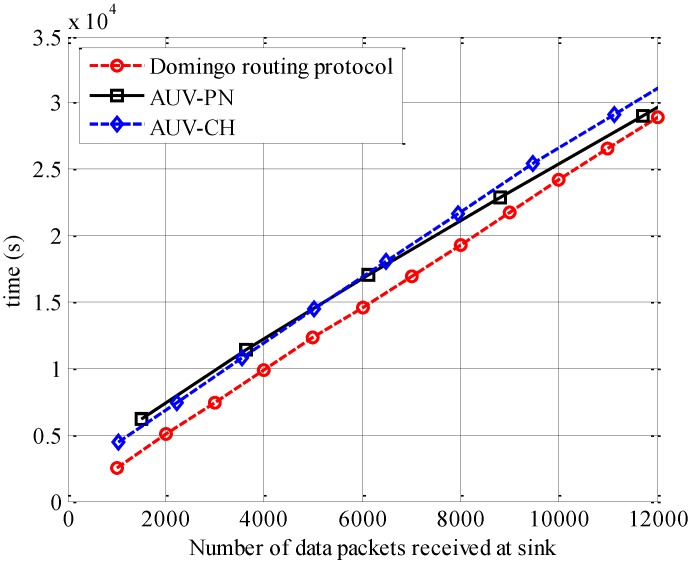
Data gathering latency *versus* number of data packets received at the sink.

Figure 11 compares the data-gathering latency, which is defined as the time taken for the number of packets gathered at the sink. It can be noted that AUV-based approaches require more time than the Domingo routing protocol to gather data at sink. This can be explained by the fact that the AUV requires time to traverse the network to gather data and then deliver it to the sink. If we compare both AUV-based approaches, it is evident that the latency for AUV-PN becomes less than that for AUV-CH as the number of packets gathered at the sink increases. For AUV-PN, it is observed that the AUV stays for a longer time in the network during a data gathering round. This results in more data being received at the sink after the completion of a data-gathering round. This increase in the number of data packets gathered at the sink reduces the effect of a longer tour time for AUV-PN, which results in lower latency compared with AUV-CH. This effect becomes more visible with higher values of data packets gathered at the sink.

**Figure 12:**
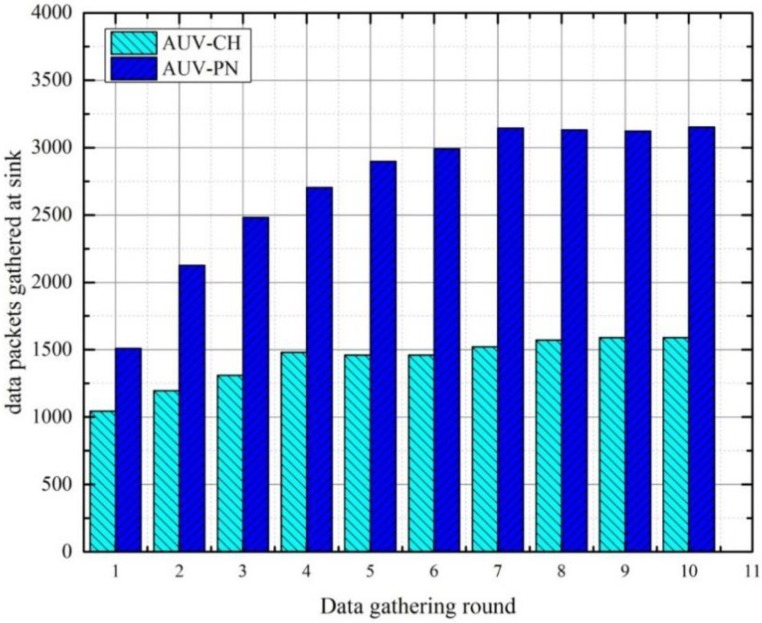
Number of data packets gathered at the sink *versus* round.

To illustrate the effect of AUV stay time in the network, we show the average number of data packet gathered at the sink for 10 rounds in Figure 12. We know that a larger AUV tour distance in a round leads to a longer MN→PN or CH data-gathering interval. Therefore, to obtain these types of results, we have set the threshold limit to *E_th_* = 500 J in order to avoid frequent changes in relay nodes which may result in large changes in the AUV tour distance with respect to the previous round. As in every round, the AUV visits each relay node in an ordered sequence; therefore, the MN→PN or CH data-gathering interval for subsequent relay nodes gradually increase which results in a higher AUV stay time at each subsequent relay node. This effect causes a steady increase in the number of data packets collected for both cases.

It is observed that the effect of a gradual increase in data-gathering interval becomes smaller as the number of rounds increases. Thus, it can be concluded that the AUV stay-time in the network achieve a mean value after certain number of round. For the AUV-CH case, the mean value of 1500 packets occurs after the fourth round, while in the case of AUV-PN the mean value of 3100 packets occurs after the seventh round. In comparison with AUV-CH, AUV-PN achieves more data collection at the sink. This is attributed to a higher number of relay nodes, which results in a higher AUV stay time.

**Figure 13:**
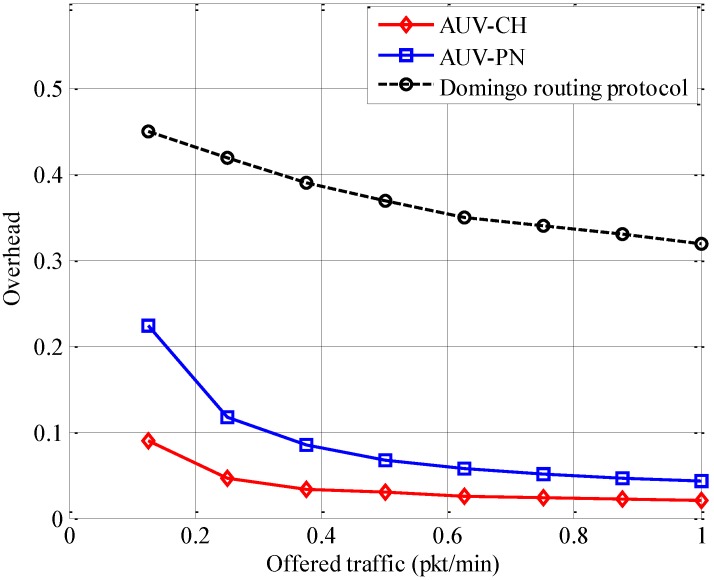
Protocol overhead.

In Figure 13, the results for protocol overhead are presented with respect to offered traffic at each node for the Domingo routing protocol, AUV-CH, and AUV-PN. The protocol overhead is defined by

(19) $$\text{overhead}=\frac{(N_{part}+N_{sch})\times L_{cont}}{N_{data}\times L_{data}}$$

where *N_part_*, *N_sch_*, and *N_data_* are the number of control packets required for network partitioning, scheduling over MN→PN and PN→AUV links, and the total number of data packets, respectively. *L_cont_* and *L_data_* are the length of control and data packet, respectively. It is observed that the Domingo routing protocol has a larger overhead than AUV-based schemes. One of the main reasons is the effect of the periodic usage of the neighbor discovery technique by the CHs. Maintenance of the route to the sink by CHs is the other main reason for such a high overhead.

It is also observed that AUV-PN has a larger overhead than AUV-CH. The additional overhead is a result of the traffic generated during PN selection in NP_Phase. In both cases, the overhead also includes the control packets required for communication between the AUV and CHs for identification of relay nodes in the cluster. For both data-gathering scenarios, the overhead gradually decreases with an increase in the offered traffic. This decrease results from the additional data packets generated at each MN, which reduces the impact of partitioning overhead more evenly. The rate of decrease becomes low because of the additional data packets gathered at each relay node, which contributes to the transmission of additional control packets at relay node→AUV links. The additional relay nodes in AUV-PN result in a higher overhead with respect to AUV-CH, but it is still much lower than the overhead generated by the Domingo routing protocol. Moreover, the additional overhead has a positive effect of uniform energy consumption over the network.

## 6. Conclusions

In this paper, we presented a distributed data-gathering scheme using an AUV over a UWSN. The proposed scheme aims at energy-constrained applications where energy consumption is the most critical parameter within an acceptable latency. In the absence of global information such as residual energy and amount of traffic to be collected, the proposed scheme organizes the network into deterministic neighborhoods, where CHs manage relay nodes, substituting them with new ones based on the residual energy at MNs. It is evident from the simulation results that the proposed scheme results in a significant reduction in energy consumption compared with the conventional AUV-based scheme (where the AUV visits only cluster heads) and the Domingo routing protocol. In addition, even though the proposed scheme produces slightly more protocol overhead than the conventional AUV-based scheme, the proposed scheme leads to more uniform energy consumption over the network, thereby increasing the lifetime of the network. 

In a deep sea networking environment, where AUV is necessary to extend the operational time of sensor nodes, the realization of an optimal deterministic neighborhood for AUV-employed data gathering is quite a challenging task because of adverse channel conditions and mobility of medium. Especially, for networks with large coverage, this task becomes more challenging owing to the variation in communication probabilities and network topologies. The proposed scheme is capable of controlling the topological changes locally by communicating such changes within the neighborhood only, thereby eliminating the requirement for global topology control over the network. This may reduce the energy consumption of sensor nodes caused by the topological changes. Thus, the proposed scheme is expected to perform well under realistic underwater networking scenarios.

In future, we will analyze and compare the proposed scheme with the existing schemes in terms of communication overhead which may be generated due to frequent network reconfiguration that results from medium mobility due to water currents and other underwater activities. We will also conduct sea experiments for different sizes of network and AUV parameters to find out the impact of different environment conditions on identified performance parameters. Furthermore, we will also look into protocol parameters such as the mobility model of the AUV, neighborhood size, and tour lengths, which are necessary for designing effective AUV employed data-gathering schemes for time-critical scenarios.
